# Alteration of growth performance, glycolipid metabolism, and bone characteristics of broiler chickens in response to different inclusion levels of dietary wheat

**DOI:** 10.5713/ab.24.0256

**Published:** 2024-08-26

**Authors:** Yilu Wang, Leilei Wang, Xuemeng Si, Yanqun Huang, Huaiyong Zhang, Wen Chen

**Affiliations:** 1 Institute of Animal Science and Technology, Henan Agricultural University, Zhengzhou, Henan, China

**Keywords:** Broiler Chickens, Growth Performance, Microbiota, Tibial Properties, Wheat

## Abstract

**Objective:**

The aim of this study was to evaluate the effects of different levels of wheat inclusion on growth performance, glycolipid metabolism, and tibial properties of broiler chickens.

**Methods:**

A total of 480 1-d-old male broiler chickens were initially fed identical starter diets until d 10. Subsequently, they were divided into 3 treatments consisting of 8 replicates with 20 birds per replicate, *i.e*., i) low-level wheat addition group, with wheat ratios of 15% and 25% during the grower and finisher periods, respectively; ii) medium-level wheat inclusion group, incorporating 30% and 40% wheat in the grower and finisher diets, respectively; and iii) high-level wheat addition group, containing 55.8% and 62.4% wheat in the grower and finisher diets, until d 39.

**Results:**

When compared to the low- and medium-level wheat diet, the high-level wheat inclusion in the diet increased feed intake and reduced the feed conversion ratio (both p<0.01), which was accompanied by a longer jejunum (p = 0.031). Meanwhile, the high-level addition of wheat displayed a decreased abundance of *Ruminococcin*, *Bacteroidetes*, and *Lactobacillus* than the low-wheat group. With the increase of the proportion of wheat treatment, the contents of cholesterol, triglyceride, and high-density lipoprotein cholesterol were elevated in serum, whereas the concentration of serum C-terminal cross-linked telopeptide of type I collagen, a bone resorption marker, was decreased. In addition, the diet with medium and high levels of wheat improved the yield load of tibia, along with comparable bone dimension and weight.

**Conclusion:**

The medium- and high-level wheat additions increased serum glycolipid deposition and enhanced tibial mechanical properties, whereas the high-level wheat diet compromised the growth performance of broiler chickens, which might be associated with the alteration of gut microbiota.

## INTRODUCTION

In livestock and poultry feed formulation, corn has become the most used feed for intensive poultry breeding due to its good palatability and high nutritional value [1]. However, the rising price of corn in recent years requires exploring alternative raw materials for corn in modern poultry production to reduce feed costs. Wheat is an important feed ingredient in poultry diets and is traditionally considered to be of very high feed value [2]. Although wheat is a valuable source of starch, protein, and some amino acids, such as methionine, lysine, and threonine, it is important to note that wheat also contains higher levels of fiber and non-starchy polysaccharides (NSP) compared to corn [3,4]. The NSP inclusion in the diet would lead to the increase of viscosity, thereby separating substrates from endogenous enzymes and decreasing nutrient utilization consequently [5]. It was confirmed that the viscosity of ileal chyme was greater in broiler chickens fed high NSP compared to low NSP [6]. In the worst-case scenario, the higher fiber could encapsulate other nutrients to shield from digestive enzymes. It thus might reduce their digestibility [7], evidenced by the poor digestibility of crude protein and ether extract in pigs fed the wheat-soybean diet as compared to those the corn-soybean diet [8,9]. Because of higher protein concentrations and faster starch digestion in wheat, however, high levels of wheat have increased crude protein content in diets [10]. Thus, it is critical to define the appropriate addition level of wheat in the diets of domestic birds.

One of the concerns is the growth performance response to wheat feeding in the meat-type industry. It was found that the addition of 40% wheat to the diet had no significant decrease in the body weight (BW) of broiler chickens [11]. Supplementation of 10% to 60% of wheat in the diets of growing-finishing pigs was also observed and it did not alter the BW and weight gain of pigs [12]. However, a compromised BW was noticed in the broiler chickens fed wheat-based diets when compared to those offered corn-based diets [13]. Moreover, it was pointed out that broiler chickens fed wheat diets possessed increased weight gain relative to the birds receiving corn-based diets [14,15]. In addition, to meet the product needs of the modern poultry industry, intestinal development, liver health, glycolipid metabolism, and bone abnormalities are often overlooked in practice. Research shows that dietary fiber can not only promote gut health by stimulating the growth of beneficial bacteria in the intestines but also aid in maintaining a healthy digestive system and support efficient nutrient absorption [16]. It was reported that wheat feeding can mediate lipid metabolism in diabetic rats [17]. A diet contained in wheat fermentation products was noticed to reduce the levels of serum triglyceride (TG) and low-density lipoprotein cholesterol (LDL-c), as well as increase the concentration of high-density lipoprotein cholesterol (HDL-c) [18]. The increased levels in serum TG, cholestenone (CHO), and LDL-c levels were also recorded after feeding refined wheat flour in model rats [19]. The alteration in glycolipid profile is closely related to bone metabolism due to the origin of both adipocytes and osteoblasts derived from the embryonic development of mesenchymal stem cells (MSCs) [20], in which osteoblasts and adipocytes are responsible for bone formation and the adipose deposition within the marrow, respectively. This reason why an inverse relationship between bone marrow adiposity and bone mass has been noticed in bone diseases such as osteoporosis [21]. A study on broiler chickens showed that leg disorders are associated with compromised growth, bone quality, and lipid metabolism [22]. It was pointed out that wheat-based feeding negatively affects the biomechanical properties of mineralization and tibia of laying hens [23]. No deleterious effects on bone mineral content were found in the diets of wheat substitution for corn in laying hens [24,25]. Considering that the goal of wheat utilization is to optimize animal health and food security rather than simple production in the broiler industry, the supplemental level of wheat for liver function, inflammatory status, and bone phenotype should be seriously reconsidered.

Therefore, the objectives of the present study were to evaluate the effects of different levels of wheat inclusion in the iso-energy and -protein diet on growth performance, glycolipid metabolism, and tibial properties of broiler chickens under the condition of supplying the multienzyme complex.

## MATERIALS AND METHODS

### Ethics and consent

All research procedures were proved by the animal care committee of Henan Agricultural University (No. HNND 20190306).

### Animal, diets, and experimental procedures

A total of 480 1-d-old male Arbor Acres (AA) broiler chickens were purchased from a local hatchery and housed in a temperature- and humidity-controlled room. The initial room temperature was set at approximately 34°C and subsequently reduced to 24°C by 20 d. The light program was 23 L:1 D during d 10 to 39. Birds were vaccinated again Newcastle disease-infectious and bronchitis on d 4. Of note, a completed random of single factor was introduced to this experiment, where birds were fed the same starter diets until 10 d and then were allocated into one of three groups based on their BW including i) the low-level wheat addition group, in which the wheat addition levels were 15% and 25% on grower and finisher periods, respectively, ii) the medium-level wheat group with the 30% and 40% of wheat in the grower and finisher periods, respectively, and iii) the high-level wheat group, where the grower and finisher diets contained 55.8% and 62.4% of wheat, respectively. Each group included 8 pens with 20 broiler chickens per pen. The diets were formulated to meet the nutrient requirements of AA broiler chickens [26] and were supplied as pellets (Table 1). The broiler chickens were weighed at d 10 and 39, and the feed intake and the number of dead or culled birds from 10 to 39 d were recorded on a replicate basis. The feed conversion rate (FCR) was expressed as the ratio of feed consumption to weight gain and the mortality was calculated in the whole trial.

### Sampling

Sampling was performed at 39 d. To ensure the consistency and minimize the variability due to sampling time, the selection of broilers, and feeding time before slaughter, the birds were fasted for 12 h and all samples were taken between 8:00 AM and 10:00 AM in the pen of bird order. In detail, one bird was selected based on the average BW of each pen, and blood was collected from the jugular vein for separating serum. After euthanasia, the liver and abdominal fat were taken and then put into a formalin solution for histology analysis. The duodenum, jejunum, ileum, and cecum were dissected, and the length and weight of intestinal segments were obtained after removing the thyme. Ileal mucosa was collected from the middle of the ileum, and stored in a sterile, RNase-/DNase-free EP tube at −80°C for gene expression determination. Enzyme-treated EP tubes were used to transfer fresh cecal contents for 16S rRNA microbiome sequencing. The tibias were immediately dissected from soft tissues and utilized to examine the bone properties of the tibia.

### Detection of serum biochemical indexes

According to the description of Lebednikaitė and colleagues [27], the serum concentrations of calcium (Ca), phosphorus (P), alanine aminotransferase (ALT), aspartate aminotransferase (AST), total protein (TP), CHO, TG, HDL-c, and LDL-c were analyzed using an automatic biochemical analyzer (Beckman Coulter AU5800; Beckman Coulter Inc., Brea, CA, USA), with corresponding commercial kits purchased from Bioengineering Institute (Nanjing, China), respectively. The concentrations of interleukin (IL)-1β, IL-6, IL-10, and tumor necrosis factor-alpha (TNF-α) were determined using commercial kits following the manufacturer’s instructions. Bone turnover markers in serum including alkaline phosphatase (ALP), procollagen type I N-terminal propeptide (PINP), and C-terminal cross-linked telopeptide of type I collagen (CTx) were quantified with available kits (Nanjing Jiancheng Bioengineering Institute, Nanjing, China).

### Liver and abdominal fat morphological changes

The fixed liver and abdominal fat samples were dehydrated with an alcohol gradient of 500, 700, 800, 950, and 1,000 mL/L and then embedded in paraffin using the HistoCore Arcadia H system (Leica, Shanghai, China). The 5-μm sections were prepared and stained with hematoxylin and eosin (H&E staining). The histopathological characteristics and changes of broiler livers and abdominal were observed with an optical microscope (Bio-Rad Laboratories, Hercules, CA, USA). The data on abdominal fat underwent quantitative processing using Image-Pro Plus (IPP 6.0; Media Cybernetics, Silver Spring, MD, USA), and the adipocyte size per treatment group was measured as the adipocyte cross-sectional surface area following the methodology described by Corino et al [28], in which fifteen adipocytes were randomly selected from the same slice field to measure their area and mean diameter.

### Tibial growth and Seedor index measurement

Referring to the methods of previous studies [29], the length and circumference of the middle part of the left tibia were measured by vernier caliper and flexible ruler, respectively. These tibias were then weighed after being dried with filter paper, and the relative tibia fresh weights were calculated as the ratio of fresh weight to BW. In addition, the Seedor index, an instructor of bone mineralization, was determined by the ratio of bone weight to length. Subsequently, the tibia was immersed in ethyl ether, air-dried at room temperature, and oven-dried at 105°C for 24 h for quantifying tibial fat-free weight.

### Bone biomechanical properties

Biomechanical testing was performed by the 3-point bending method with a TA-XT Plus Texture Analyzer (TA. XT. Plus; Stable Micro Systems, Surrey, UK) at a constant 50 kg load cell according to the previous description [29] Loading proceeded at the mid-point of the right tibia with a constant rate (5 mm/min) up to the breaking of the bone. Force-displacement data were obtained to calculate bone stiffness, yield load, ultimate strength, and area-under-the-curve (AUC), in which the yield point and stiffness were defined as the load at which the load-deformation relationship ceased to be linear and the slope of the linear portion of the load-displacement curve.

### 16S rRNA sequencing of cecal microbiome

The total genomic DNA was extracted from cecum digesta, followed by quality evaluation with electrophoresis using 0.8% agarose gels. The DNA concentration was quantified with a Tecan F200 microplate reader (Infinite F200; Tecan, Männedorf, Switzerland). The V4 region of the *16S rRNA* gene was then amplified using specific primers (F: 5′-CCTA CGGGRSGCAGCAG-3′; R: 5′-GGACTACVVGGGTATC TAATC -3′). The polymerase chain reaction (PCR) products were purified and sequenced on the Illumina MiSeq platform. The two-ended sequences were spliced using FLASH [30]. Each sample sequence was then isolated from raw reads based on the barcode using QIIME (version 1.9.0), with the Barcode sequence truncated. The UPARSE algorithm was used to cluster the operational taxonomic units at a 97% threshold value. The alpha diversity was estimated with the Chao 1, Simpson, and Shannon indexes and Observed species, and beta diversity at the genus level was estimated by calculating Bray-Curtis dissimilarity and visualized with principal coordinates analysis (PCoA). The microbial taxa that differed between the different groups were identified using linear discriminant analysis (LDA) with a threshold of 2.0.

### Statistical analysis

Statistical analysis was carried out using IBM SPSS statistic 26 (Statistical Packages for Social Science 26; IBM, Corporation, Armonk, NY, USA). Data was checked for normality via the Shapiro-Wilk test. One-way analysis of variance with Tukey’s test or Kruskal-Wallis with Dunn’s test for normally or non-normally distributed data, respectively, were used to evaluate the statistical differences of biological parameters. Pearson’s correlation analysis was performed to determine the correlation between tibia ultimate strength and lipid metabolism. The alterations in microbial composition following 16S rRNA amplicon sequencing were determined in R using the Microbiota Process package for community analysis (version 3.17). A statistically significant and tendency was considered when a p<0.05 and p≤0.10, respectively.

## RESULTS

### Growth performance

The effects of different levels of addition with wheat on the growth performance of broiler chickens are presented in Table 2. In comparison to the low-level wheat group, the increased wheat addition tended to reduce the BW (p = 0.059) at 39 d and weight gain (p = 0.050) from 10 to 39 d, whereas the high-level wheat group showed a significant increase in feed consumption and decrease in FCR as compared to the low- and medium-level wheat diets (both p<0.001). Of note, the incorporation of wheat in diets did not significantly change the mortality during 1 to 39 d.

### Alteration in the gastrointestinal tract

**Figure 1:**
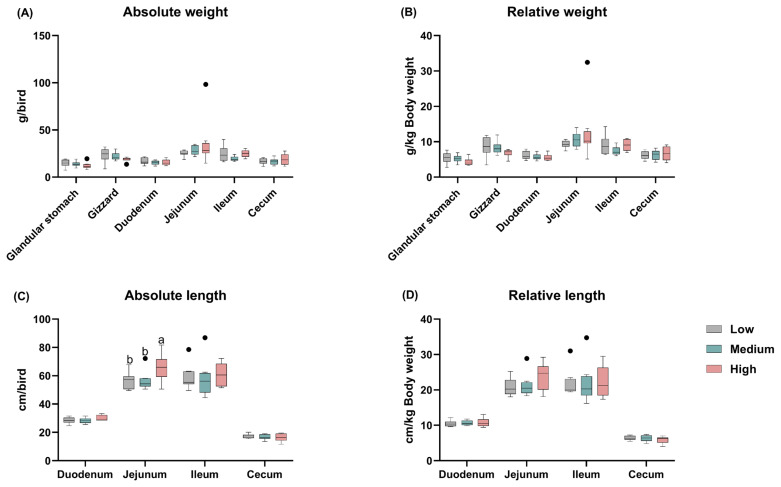
Effects of different levels of dietary wheat on the digestive tract index in 39-d-old broiler chickens. (A) Absolute weight of glandular stomach, gizzard, small intestine, and cecum. (B) Relative weight of glandular stomach, gizzard, small intestine, and cecum. (C) Absolute length of small intestine, and cecum. (D) Relative length of small intestine, and cecum. Values are means and standard deviation represented by vertical bars in a scatter plot. In the box-whiskers plots, boxes are bounded by the 25th and 75th percentiles, with the median shown by the line bisecting the box. Whiskers extend to the full range of the data. Outliers are represented by dots. ^a,b^ Different letters represent the significant difference at p<0.05 (n = 8).

The effect of dietary treatments on digestive organ development is presented in Figure 1. Different levels of wheat supplementation in the diets of broiler chickens have little effect on the weight of glandular stomach, gizzard, small intestine, and cecum. Meanwhile, the length of the intestine was examined, and found the dietary wheat supplementation had not apparently changed the absolute and relative length of the duodenum, ileum, and cecum, but the birds fed the high-level wheat diet exhibited a significantly longer jejunum when compared to those fed the low- and medium-levels wheat diets (p = 0.031; Figure 1C).

### Cecal microflora

**Figure 2:**
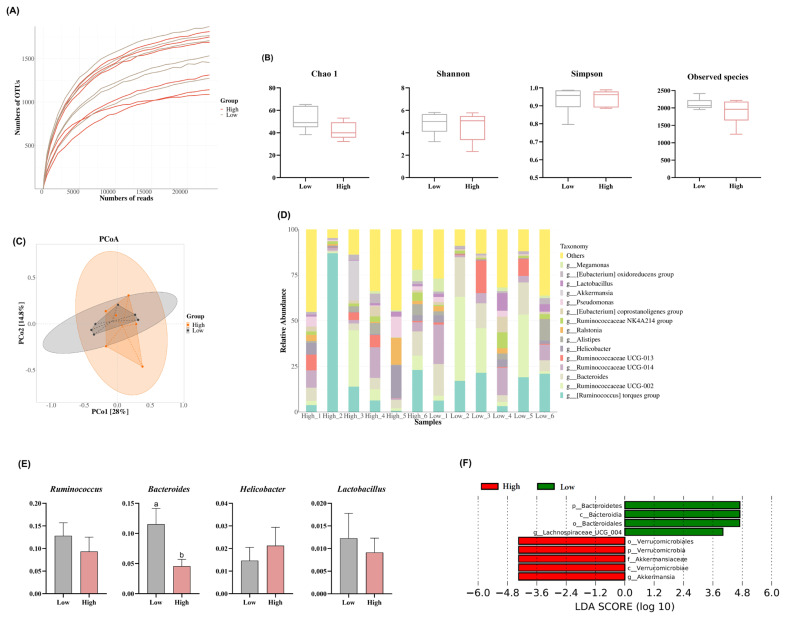
Effects of different levels of dietary wheat on the cecal microbiota in 39-d-old broiler chickens. (A) The dilution curve showed the trend of species richness with sequencing depth. (B) Species alpha diversity and richness were evaluated by Chao1, Shannon, Simpson and Observed species indices. (C) Principal coordinate analysis plot (PCoA) of cecum microbiome diversity based on Bray-Curtis distance. (D) Composition of microbiota at genus level in cecum contents of broilers. (E) Abundance of *Ruminococcus*, *Bacteroides*, *Helicobacter*, and *Lactobacillus*. (F) Cladogram of the significant differential taxa enriched in the corresponding treatment. Values are means and standard deviation (n = 6). ^a,b^ Different letters represent the significant difference (p<0.05).

**Figure 3:**
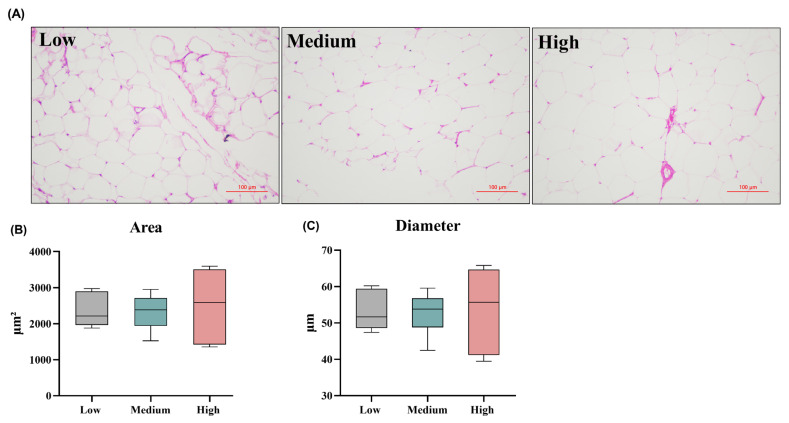
Effects of different levels of dietary wheat on the histopathological changes of adipose tissue in 39-d-old broiler chickens. (A) Representative images of adipocyte tissue in three processing groups (hematoxylin and eosin staining, 200×). (B and C) Quantification of adipocyte cell diameter and adipose area using the Image Pro Plus software. Values are means and standard deviation represented by vertical bars in a scatter plot. In the box-whiskers plots, boxes are bounded by the 25th and 75th percentiles, with the median shown by the line bisecting the box. Whiskers extend to the full range of the data.

Considering the significantly increased feed intake and jejunal length, as well as the compromised FCR due to the addition of high-level wheat in the diet, the alterations in cecal microbiota between the low- and high-level wheat groups were determined and showed that the flora richness of each sample increased with the increase of sequencing depth, and the end of the curve tended to be flat, indicating that the amount of sequencing data was reasonable (Figure 2A). There were no significant differences between the low- and high-level wheat groups in the alpha diversity, evidenced by comparable Observed species, Chao1 (p = 0.08), Shannon, and Simpson indexes (Figure 2B). PCoA showed a clear separation between the two groups (Figure 2C), where PCoA1 and PCoA2 accounted for 28% and 14.8% of the variation in microbial diversity, respectively. At the genus level, the high-level wheat diet decreased the abundance of *Ruminococcin*, *Bacteroidetes*, and *Lactobacillus*, whereas it increased the proportion of *Helicobacter* as compared to the low wheat diets group (Figure 3D, 3E). The LDA showed significant enrichment of *Bacteroidetes* in the low-level wheat group and *Verrucomicroblale* in the high-level wheat group in the cecal microbiota (Figure 2F).

### Lipid metabolism

When compared with the low- and medium-level wheat diets, the number of fat cells in the high-level wheat diet was significantly higher in the same field of view (Figure 3A). However, there was no significant effect on the adipocyte area, and mean diameter at different levels of wheat treatment (Figure 3B, 3C). With the increased wheat levels in the diets, the contents of CHO, TG, and HDL-c in serum were significantly increased (p<0.05). Meanwhile, the serum concentrations of GLU and LDL-c were similar among the experimental groups (Table 3).

### Bone properties

**Figure 4:**
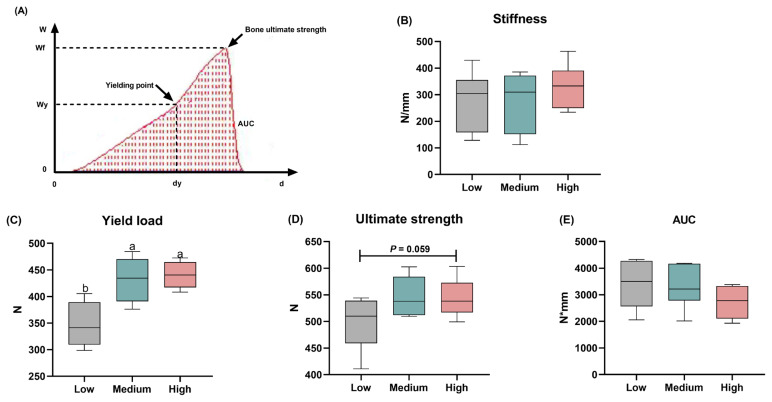
Effects of different levels of dietary wheat on the mechanical properties of tibia in 39-d-old broiler chickens. (A) The displacement of tibia at the stress point measured in the process of loading changes with the applied load. (B) Bone stiffness that expressed as the slope of the linear portion of the load-displacement curve (N/mm). (C) Yield load (N). (D) Ultimate load (N). (E) Area under the load-displacement curve (AUC, work to failure, N×mm). Values are means and standard deviation represented by vertical bars in a scatter plot. In the box-whiskers plots, boxes are bounded by the 25th and 75th percentiles, with the median shown by the line bisecting the box. Whiskers extend to the full range of the data. ^a,b^ Different letters represent the significant difference at p<0.05 (n = 8).

The effects of different levels of addition with wheat on the bone properties of broiler chickens are presented in Table 4. The experimental treatments failed to affect the length and circumference of the tibia. The supplementation of wheat neither changed fresh weight, relative weight, nor fat-free weight in this study. There were no significant differences between the three groups regarding the Seedor index (Table 4). Moreover, the bone mechanical parameters including yield load, ultimate strength, stiffness, and AUC were assessed according to Figure 4A, and the results showed that the stiffness was not changed by dietary wheat supplementation (Figure 4B). The yield load (p<0.05) and ultimate strength (p = 0.059) of the tibia were elevated by the increased wheat levels (Figures 4C, 4D). However, no apparent differences were found regarding AUC among the three groups (Figure 4E).

### The correlation between bone strength and serum glycolipid

**Figure 5:**
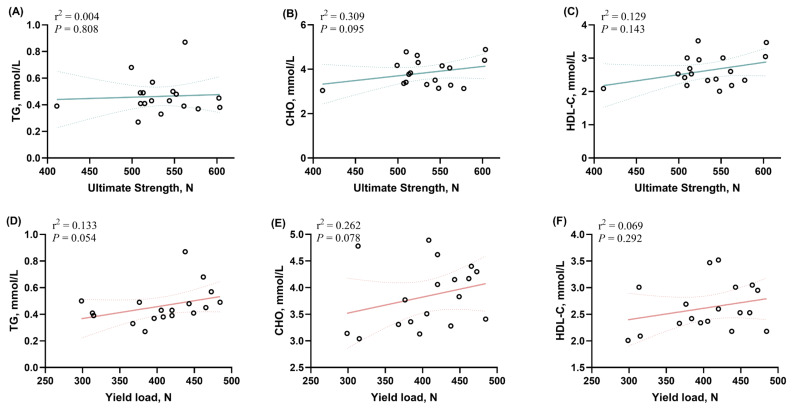
Analysis of the correlation between the ultimate strength and yield load of tibial bone and blood lipid content in broiler. (A–C) The correlation between the ultimate strength and triglyceride (TG), cholestenone (CHO), and high-density lipoprotein cholesterol (HDL-c), as well as (D–F) The correlation between the yield load and TG, CHO, HDL-c were performed by Pearson’s correlation analysis.

To evaluate the relationship between bone ultimate strength, yield load, and serum glycolipid levels, a Pearson’s correlation matrix was performed and showed that the ultimate strength of bone tended to positively correlate with serum CHO content (p = 0.095). Similarly, a trend between yield load and serum TG (p = 0.043) and CHO (p = 0.078) was also observed. No other significant changes were noted in the determined parameters (Figure 5).

### Bone turnover

**Figure 6:**
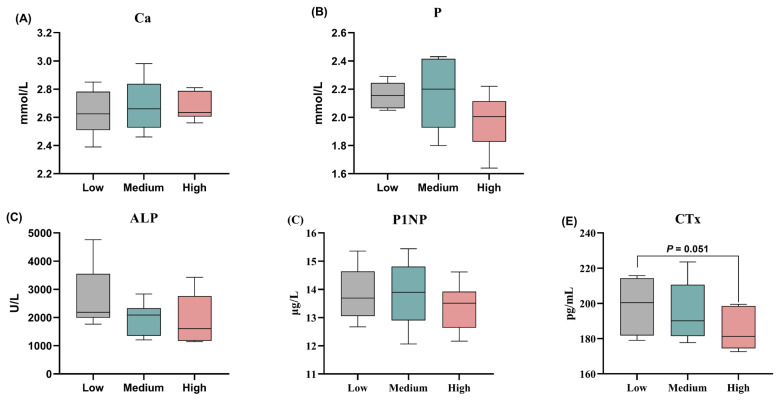
Effects of different levels of dietary wheat on the bone metabolism in 39-d-old broiler chickens. The content of serum (A) calcium (Ca); and (B) phosphorus (P), bone formation markers including; (C) alkaline phosphatase (ALP); and (D) procollagen type I N-terminal propeptide (PINP), as well as C-terminal cross-linked telopeptide of type I collagen (CTx), a bone resorption instructor, were quantified using commercial kits. Values are means and standard deviation represented by vertical bars in a scatter plot. In the box-whiskers plots, boxes are bounded by the 25th and 75th percentiles, with the median shown by the line bisecting the box. Whiskers extend to the full range of the data.

There was no significant effect on serum Ca and P at different levels of wheat treatment (Figures 6A, 6B). The outcomes of serum bone formation markers revealed that the concentration of P1NP and ALP were similar among the three wheat diets (Figures 6C, 6D). Nevertheless, Serum CTx level, representing bone resorption, was decreased by dietary high-level wheat diet when compared with the low-level wheat group (p = 0.051; Figure 6B).

### Liver health and serum inflammatory factor

**Figure 7:**
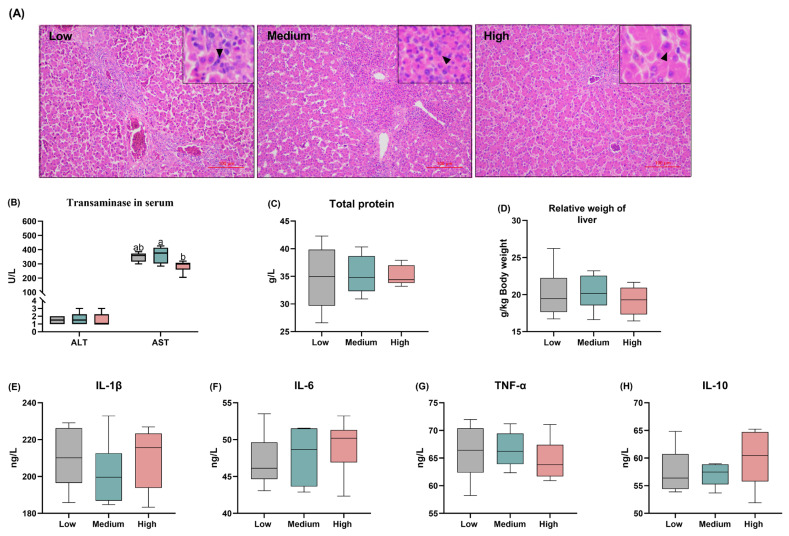
Effects of different levels of dietary wheat on the liver function and systemic inflammation in 39-d-old broiler chickens. (A) Micrograph of liver microstructure (hematoxylin and eosin staining, 200×). The small black arrow indicates inflammatory cell infiltration. (B) Serum level of transaminase including alanine aminotransferase (ALT) and aspartate aminotransferase (AST); (C) Serum total protein content. (D) The relative weight of liver. The content of inflammatory factors interleukin (IL)-1β, IL-6, tumor necrosis factor-alpha (TNF-α), and IL-10 in serum. Values are means and standard deviation represented by vertical bars in a scatter plot. In the box-whiskers plots, boxes are bounded by the 25th and 75th percentiles, with the median shown by the line bisecting the box. Whiskers extend to the full range of the data. ^a,b^ Different letters represent the significant difference at p<0.05 (n = 8).

Effects of dietary treatments on liver microstructure of broiler chickens Figure 7A, the histological analysis showed normal hepatocytes without necrosis. The cells were arranged in order, and the nuclei were large and round with even cytoplasm across all treatment groups. With the increase in dietary wheat content, the inflammatory cell infiltrating area in liver tissue decreased, the hepatocyte boundary increased, and the number of neutrophils decreased in the high-level wheat group. Reflection on the serum biochemical indexes, the high-level wheat diet decreased serum AST activities when compared with the medium-level wheat group. There were no significant differences in terms of the levels of ALT and TP, as well as the relative of the liver among the three groups (Figures 7B–7D). In addition, the outcomes of serum inflammatory factor revealed that the dietary wheat inclusion did not change the anti-inflammatory cytokine IL-10 and pro-inflammatory factors IL-1β, IL-6, and TNF-α concentration in the present study (Figures 7E–7H).

## DISCUSSION

### The effects of various wheat levels on growth performance

With the rapid development of poultry farming, the shortage of global supply driving the rising price of corn demands seeking its replacement in the animal husbandry. Wheat has been widely used as energy feed in animal husbandry, especially in Europe, implying wheat has become the primary choice to replace corn. However, the influences of wheat-based diets on BW or weight gain are inconclusive. The improved weight gain in the broiler chickens fed wheat diets was observed as compared to those receiving corn-based diets [14,15]. The positive role might be attributed to the corporation of insoluble NSP in ingredients or the higher weight mainly derived from the visceral organs [15]. No apparent effects on the BW were also found in broiler chickens fed an inclusion of 40% wheat in a corn-based diet [11] and growing-finishing pigs that consumed a corn-soybean meal-based diet with 10% to 60% of wheat [12]. In this study, the increased wheat levels in the diet led to a decline in BW and weight gain, which was confirmed in a previous study indicating that the wheat-based diets compromised the BW of broiler chickens when compared to those offered the corn-based diets [13]. It was reported that a diet enriched in NSPs, such as wheat and barley, could increase the thyme viscosity, reducing feed consumption and growth performance [31]. In comparison to the corn-based diet, a wheat-based one caused a 3 times increase in viscosity of ileal digesta in broiler chickens [6]. Therefore, the delayed transit time and gut motility due to higher viscousity hindering feed intake is warranted in higher NSP-contained diets [5], including the wheat-based diet. A study on broiler chickens found that the supplementation of a wheat-based diet impaired the feed intake and reduced the ratio of feed consumption to weight gain from 25 to 42 d in broiler chickens [11]. In accordance with previous research on birds [14], the feed consumption of birds in high-wheat diets was higher than those fed other diets in the current study, thereby bringing about an inferior FCR. The possible explanation includes the palatability of wheat to animals [32] and the supplementation of enzymes [33]. Of note, incorporating NSP-degrading enzymes, particularly xylanase, into wheat-based diets could damage fiber structures, resulting in a decrease in intestinal viscosity and subsequently increasing nutrient digestibility of birds and promoting foraging [34,35]. Collectively, under the condition of supplementing exogenous enzymes, the medium-level wheat inclusion did not obviously decrease growth performance, when the supplemented level of wheat extended to 57.77% and 62.38% in grower and finisher diets, respectively, the wheat treatment increased feed intake and compromised FCR of broilers chickens.

### The response of intestinal development and cecal microbiota to the different levels of wheat

To uncover the mechanism underlying the supplemented levels of wheat on the growth performance of broiler chickens, the development of gastrointestinal tract and cecal microbiota were detected. It was pointed out that wheat bran contains a lot of insoluble dietary fiber, which has been shown to stimulate gizzard development [36]. The wheat diet increased the weight of the cecum, and the length of the duodenum and ileum as compared to the corn diet [37]. Analogously, an absolute longer jejunum was noticed in the high proportion of wheat group than that in other groups in the present study. Moreover, the composition of gut microbiota is closely associated with the digestion and absorption of nutrients, immune, and intestinal barrier functions of animals, and it is also influenced by the diet type [15,38]. The crude fiber contained in wheat could be directly fermented by microbiota as the energy material, reflecting that the abundance of species of gut microbiota depends on the dietary fiber types and levels [39]. Incorporation of corn-based diets, and the supplementation of wheat-based diets increased the account of *Escherichia coli*, a well-known harmful bacterium [11], and decreased the proportion of *Ruminococcus* in the caeca microbiota profile of broiler chickens [6]. A study on growing pigs found that the feeding of wheat-based diets decreased the levels of *Escherichia-Shigella* and increased the proportion of beneficial bacteria such as *Bifidobacterium* and *Lactobacillus* at the genus level [38]. The discrepancy among these researchers may imply that the alterations of microbial composition rely on the dietary components, trial objects, environment, *etc*. In this study, the high wheat addition decreased the abundance of *Ruminococcin*, *Bacteroidetes*, and *Lactobacillus*, whereas it increased the proportion of *Helicobacter* as compared to the low wheat groups. *Bacteroides* play important roles in the degradation of polysaccharides and oligosaccharides [40,41]. *Lactobacillus* is widely distributed in domestic birds, and oral supplementation with *Lactobacillus* improved gut microbiota and stimulated intestinal improvement of broiler chickens, thereby promoting growth performance [42,43]. On the contrary, *Helicobacter*, a gram-negative bacterium, is often found to cause inflammation and ulceration [44]. Therefore, the decreased abundance of *Bacteroidetes* and *Lactobacillus*, along with increased the proportion of *Helicobacter* in the high-level wheat diet might result in compromised growth performance.

### The alteration in glycolipid metabolism and bone characteristics of broilers response to the supplemented levels of wheat

Wheat serves as an energy source for broiler chickens and has a higher level of starch [3]. Previous studies have found that the supplementation of wheat fermentation products caused the reduction of serum TG and LDL-c and resulted in increased HDL-c [18]. Nevertheless, other outcomes suggested that serum TG, TC, and LDL-c levels were increased significantly after feeding refined wheat flour for 16 weeks, which was accompanied by decreased HDL-c levels in model rats [19]. The results are similar to the data in this experiment, i.e., the increase of wheat substitution levels increased the contents of CHO, TG, and HDL-c in serum, indicating this increased fat deposition. H&E staining also showed an increase in the size of adipocytes in the high group. These results suggest that higher levels of wheat in broiler feed can lead to an increase in fat accumulation.

Of note, a link between lipid metabolism and bone turnover was observed. Guo et al [22] showed that leg disorders are associated with compromised growth, bone quality, bone structure, and lipid metabolism. The probable mechanism might be partly due to the common origin of adipocytes and osteoblasts, both originating from MSCs [20]. MSCs present in bone marrow possess the ability to differentiate into osteoblasts and adipocytes, which play a crucial role in bone formation and the adipose tissue component within the marrow, respectively. In this study, the correlation analysis of serum glycolipid profile and bone properties in broiler chickens showed a linear correlation between serum CHO and both ultimate strength and yield load of tibia, the content of TG levels also positively related to tibial yield load. In this context, an inverse relationship between bone marrow adiposity and bone mass has been noticed in bone diseases such as osteoporosis [21]. These data suggested that it is necessary to further explore the dynamic relationship between MSCs, osteoblasts, and adipocytes for revealing bone metabolism and adipose tissue development.

In this study, feeding the medium- and high-level wheat increased the yield load and ultimate strength of the tibia, suggesting that the diets contained appropriate levels of wheat combining with complex enzymes could enhance the mechanical properties of the tibia of broiler chickens. The positive effects of wheat addition on tibia are mainly because of the enzyme addition. Research by Olgun et al [23] found that wheat-based feeding negatively affects the biomechanical properties of mineralization and tibia of hens but adding enzymes decreases this negative effect of wheat. The replacement of corn with wheat in laying diets without affecting the quality of the eggshell or bone mineral content, can improve the strength and quality of the bones after supplementation with xylanase in these diets [24,25]. Similarly, Kiarie et al [14] reported decreased phosphorus retention in hens fed on wheat-based diets, but increased phosphorus retention in broiler chickens with enzymes (xylanase) added to feed. In this study, different levels of wheat had no significant effect on the levels of serum Ca and P in broiler chickens. Considering the bone turnover, serum PINP and ALP levels, both secreted by osteoblasts, can be used as an indicator of new bone formation [45]. In this experiment, using different levels of wheat in the diet had no significant effect on the serum content of P1NP and ALP in broiler chickens. CTx is a marker of bone resorption, and its level reflects the bone resorption activity of osteoclasts [46]. The high-level wheat substitution group resulting in decreased CTx content implied that the positive roles of wheat administration on tibial strength might partially be attributed to reduced bone absorption.

### The effects of varying wheat levels on hepatic function

It is well-established that the goal of animal husbandry is to optimize animal health and food security rather than simple production, therefore, the liver function and inflammatory status were seriously reconsidered. By evaluating the healthy status of hepatocytes and systemic inflammation, the present data suggests that the supplementation of high-level wheat did not impair liver function, evidenced by decreased AST activity, a biomarker of early liver injury, and similar serum levels of ALT, TP, and inflammatory cytokines in this study. Moreover, the high levels of wheat inclusion could reduce liver inflammatory cell infiltration in broiler chickens, which was consistent with previous findings that wheat malt extracts (100 or 200 mg/kg) reduce the degree of hepatological histological injury and serum ALT and AST in a dose-dependent manner [47]. Therefore, in this experiment, the dose of wheat in the diets of broiler chickens had no deleterious effects on liver health and inflammatory status.

## CONCLUSION

In summary, under the condition of supplementing exogenous enzymes, the inclusion of a high-level wheat in the iso-energy and -protein diet (55.8% and 62.4% in grower and finisher diets, respectively) increased feed intake and impaired FCR, which might be associated with the alteration of gut microbiota such as the decreased abundance of *Bacteroidetes* and *Lactobacillus*. Moreover, different levels of wheat in the diets of broiler chickens have little effect on hepatocyte function, whereas the serum glycolipid deposition and tibial mechanical properties were improved by the supplementation of wheat (less than 62.4%) in broiler chicken.

## Figures and Tables

**Table 1:** Ingredients and calculated analysis of nutrient in the basal diet (as-fed)

Items	Starter diet (1 to 10 d)	Grower diet (11 to 21 d)			Finisher diet (22 to 39 d)		
	
		Low	Medium	High	Low	Medium	High
Ingredients (%)							
Corn	35.18	35.18	22.27	0	32.34	19.28	0
Wheat	15.00	15.00	30.00	55.77	25.00	40.00	62.38
Wheat flour	15	15	15	15	10	10	10
Soybean oil	1.0	1.0	1.1	1.3	3.9	4.1	4.2
Soybean meal	20.2	20.2	18.0	14.2	16.0	13.8	10.5
Peanut meal	3	3	3	3	3	3	3
Corn gluten meal	3	3	3	3	3	3	3
Meat meal	2.2	2.2	2.2	2.2	2.2	2.2	2.2
Sodium chloride	0.20	0.20	0.20	0.20	0.25	0.25	0.25
Limestone	0.98	0.98	0.95	0.90	1.07	1.04	1.00
Montmorillonite	0.2	0.2	0.2	0.2	0	0	0
Dicalcium phosphate	1.60	1.60	1.57	1.55	0.80	0.80	0.77
Choline	0.1	0.1	0.1	0.1	0.08	0.08	0.08
L-lysine HCl	0.90	0.90	0.95	1.08	1.02	1.10	1.20
DL-Methionine	0.22	0.22	0.22	0.23	0.20	0.20	0.21
L-Threonine	0.25	0.25	0.27	0.30	0.27	0.28	0.34
Complex enzyme^1)^	0.3	0.3	0.3	0.3	0.3	0.3	0.3
Sodium bicarbonate	0.1	0.1	0.1	0.1	0.1	0.1	0.1
Sodium butyrate	0.04	0.04	0.04	0.04	0.04	0.04	0.04
Premix^2)^	0.53	0.53	0.53	0.53	0.53	0.53	0.53
Total	100	100	100	100	100	100	100
Nutrient content (%)							
AME (Kcal/kg)	2,950	2,950	2,950	2,950	3,150	3,150	3,150
Crud protein^3)^	21.32	21.31	21.35	21.44	20.31	20.30	20.35
Dry matter^3)^	86.82	86.82	87.23	87.94	87.16	87.57	88.18
Crud fiber	2.50	2.50	2.45	2.35	2.34	2.28	2.20
Calcium^3)^	0.88	0.88	0.88	0.88	0.61	0.61	0.63
Total phosphorus^3)^	0.67	0.67	0.68	0.68	0.50	0.49	0.49
Available phosphorus	0.45	0.45	0.45	0.45	0.38	0.38	0.38
Dig. Lysine	1.28	1.28	1.28	1.28	1.23	1.25	1.24
Dig. Methionine	0.5	0.5	0.48	0.51	0.45	0.45	0.46
Dig. Tryptophan	0.19	0.20	0.20	0.21	0.17	0.18	0.18
Dig. Threonine	0.86	0.85	0.86	0.84	0.81	0.81	0.84

1) 100 g complex enzyme include: 2% Xylanase (100,000 IU/g); 0.75% cellulase (8,000 IU/g); 0.8% β-glucanase (50,000 IU/g); 0.3% mannase (50,000 IU/g); 0.5% Pectinase (30,000 IU/g); 0.5% α-galactosidase (2,000 IU/g).

2) Premix providing per kg of diet: vitamin A (retinyl acetate), 9,985 IU; vitamin D_3_ (cholecalciferol), 4,293 IU; vitamin E (dl-α-tocopherol acetate), 39.3 mg; vitamin K_3_ (menadione), 3.975 mg; vitamin B_1_ (thiamine), 4.293 mg; vitamin B_2_ (riboflavin), 9.67 mg; niacin, 62.96 mg; D-pantothenic acid, 21.75 mg; vitamin B_6_ (pyridoxine-HCl), 4.60 mg; vitamin B_12_ (cyanocobalamine), 0.038 mg; folic acid, 2.16 mg; biotin, 0.2 mg; Fe (FeSO_4_·H_2_O), 41.44 mg; Cu (CuSO_4_·5H_2_O), 10 mg; Zn (ZnO), 80.5 mg; Mn (MnO), 85.56; (Ca(IO_3_)_2_), 0.992 mg; Se (Na_2_O_3_Se), 0.304 mg.

3) These values were measured value.

AME, apparent metabolizable energy; Dig., digestible.

**Table 2:** Effects of different levels of dietary wheat on growth performance in broiler chickens (10 to 39 d of age)

Item	Low	Medium	High	SEM	p-value
BW at 10 d (g/bird)	285	285	285	6.69	0.996
BW at 39 d (g/bird)	2,531	2,424	2,444	97.57	0.059
BW gain10 to 39 d (g/bird)	2,246	2,139	2,159	94.92	0.050
FI during 10 to 39 d (g/bird)	3,440^b^	3,431^b^	3,763^a^	198.71	<0.001
FCR during 10 to 39 d (g/g)	1.53^b^	1.61^b^	1.74^a^	0.12	<0.001
Mortality (%)	5.13	6.25	5.00	6.12	0.877

a,b Different letters in the same row represent the significant difference (p<0.05, n = 8).

SEM, standard error mean; BW, body weight; FI, feed intake; FCR, feed conversion ration expressed as the ratio of FI to BW.

**Table 3:** Effects of different levels of dietary wheat on serum glycolipid metabolism in 39-d-old broiler chickens

Item	Low	Medium	High	SEM	p-value
GLU (mmol/L)	13.65	13.27	13.25	0.83	0.668
CHO (mmol/L)	3.31^b^	3.77^b^	4.39^a^	0.55	<0.001
TG (mmol/L)	0.39^b^	0.43^ab^	0.57^a^	0.14	0.048
HDL-c (mmol/L)	2.27^b^	2.57^ab^	2.94^a^	0.44	0.018
LDL-c (mmol/L)	0.93	0.99	0.92	0.21	0.728

a,b Within a row, values with different superscripts indicate a significant difference (p<0.05, n = 8).

SEM, standard error mean; GLO, glucose; CHO, cholesterol; TG, triglyceride; HDL-c, high-density lipoprotein cholesterol; LDL-c, Low-density lipoprotein cholesterol.

**Table 4:** Effects of different levels of dietary wheat on bone properties in 39-d-old broiler chickens

Item	Low	Medium	High	SEM	p-value
Length (cm)	9.90	9.68	9.87	0.34	0.403
Circumference (cm)	2.84	2.66	2.79	0.26	0.374
Fresh weight (g)	13.02	12.30	13.20	2.22	0.710
Relative weight (% body weight)	0.48	0.46	0.47	0.05	0.859
Fat-free weight (g)	5.72	5.90	5.99	0.86	0.826
Seedor (g/cm)	1.32	1.27	1.33	0.21	0.748

SEM, standard error mean.
